# Histone methylation regulates Hif‐1 signaling cascade in activation of hepatic stellate cells

**DOI:** 10.1002/2211-5463.12379

**Published:** 2018-01-25

**Authors:** Fei Hong, Lu Wan, Jie Liu, Ke Huang, Zhenmeng Xiao, Yingjing Zhang, Chunwei Shi

**Affiliations:** ^1^ Department of Pathogen Biology School of Basic Medicine Huazhong University of Science and Technology Wuhan China

**Keywords:** hepatic stellate cells, Hif‐1, histone methylation

## Abstract

Liver fibrosis is characterized by deposition of excessive extracellular matrix (ECM). The major source of ECM is activated hepatic stellate cells (HSCs). Previously, we reported that hypoxia‐inducible factor‐1 (Hif‐1) regulates activation of HSCs through autophagy. In current work, human HSC cell line LX‐2 was used as cell model. It was determined that trimethylation of H3 histone on lysine 4 (H3K4me3) occurred in the Hif‐1 transcriptional complex. Inhibition of modifications of histone methylation suppressed Hif‐1 nuclear transport, autophagosome formation, and activation of LX‐2 cells. These data suggest that histone methylation modification plays an important role in the Hif‐1 signaling cascade regulating HSC activation.

**Abbreviations**
BNIP3: BCL2/adenovirus E1B 19 kDa interacting protein 3
GLUT‐1: glucose transport protein‐1
GSY1: glycogen synthase 1
H3K27: histone H3 lysine 27
H3K36: histone H3 lysine 36
H3K79: histone H3 lysine 79
H3K9: histone H3 lysine 9
H3R17: histone H3 arginine 17
H3R26: histone H3 arginine 26
H3R2: histone H3 arginine 2
H4K20: histone H3 lysine 20
H4R3: histone H3 arginine 3
Hif‐1: hypoxia‐inducible factor‐1
Hif‐1α: Hif‐1 oxygen‐sensitive alpha subunit
Hif‐1β: Hif‐1 constitutive beta subunit
HSCs: hepatic stellate cells
KDM: lysine (K)‐specific demethylase
me1: methylation
me2: dimethylation
me3: trimethylation
OGT: O‐linked *N*‐acetylglucosamine (GlcNac) transferase
P4HA1: prolyl 4‐hydroxylase, alpha polypeptide I
STC1: Stanniocalcin‐1
α‐SMA: α‐smooth‐muscle actin

Liver fibrosis is a worldwide health issue due to the lack of effective treatment. All kinds of acute or chronic liver injuries, including viral hepatitis, nonalcoholic steatohepatitis, alcohol‐induced liver damage, parasitemia, and autoimmune diseases, result in liver fibrosis and the end stage of liver fibrosis, cirrhosis 1, 2. Liver fibrosis is a reversible wound‐healing process, which is characterized by deposition of excessive extracellular matrix (ECM) 3. The major source of ECM is activated hepatic stellate cells (HSCs), which plays an important role in pathogenesis of liver fibrosis 4. Activated HSCs are always accompanied by cytoskeleton reconstruction, expression of α‐smooth‐muscle actin (α‐SMA) and vimentin, and loss of lipid droplets, which together facilitate the development of liver fibrosis 5, 6, 7.

Hypoxia‐inducible factor‐1 (Hif‐1) is a heterodimeric transcription factor consisting of an oxygen‐sensitive alpha subunit (Hif‐1α) and a constitutive beta subunit (Hif‐1β) that promotes cell survival by regulating the expression of essential genes under oxygen deprivation 8, 9. We previously reported that hypoxia‐inducible factor‐1 (Hif‐1) acts as a main regulator in the activation of HSCs 5 and Hif‐1 regulates autophagy to activate HSC 6; however, the specific molecular mechanism is still vague.

Recently, the role of post‐translational modification in gene regulation has been paid attention. Post‐translational modification refers to methylation, acetylation, ubiquitination, phosphorylation, and SUMOylation occurred in histone of nucleosome, which is one kind of epigenetic modification resulting from changes in gene expression and functions without alterations in the DNA sequence 10. Nucleosomes are the basic unit of chromatin. Each of the two H2A, H2B and H3 and H4 subunits form a histone octamer and the 146 bp of DNA surrounds the histone octamer to form nucleosomes. Histone methylation modification, one kind of post‐translational modification, commonly occurs in lysine or arginine residues of the N terminus of H3 and H4 histone in nucleosome. Lysine methylation usually occurs in H3 lysine 4 (H3K4), lysine 9 (H3K9), lysine 27 (H3K27), lysine 36 (H3K36), lysine 79 (H3K79), and H4 lysine 20 (H4K20). Arginine methylation usually occurs in H3 arginine 2 (H3R2), arginine 8 (H3R8), arginine 17 (H3R17), arginine 26 (H3R26), and H4 arginine 3 (H4R3). Depending on the number of methylated groups on the residue, they are divided into methylation (me1), dimethylation (me2), or trimethylation (me3). Among them, H3K4me3, H3K4 trimethylation, was found to especially exhibit high abundances near the transcriptional start site on promoter region of activated gene 11. In recent years, the relatedness of hypoxia and histone methylation is gradually being recognized in cellular signal integration, which indicated that alteration of epigenetic homeostasis plays a role in gene regulatory switches under hypoxia 12, 13.

In current work, using genomewide expression chip analysis, up‐regulation of several genes regulating histone methylation was determined in hypoxia‐induced HSC line, LX‐2. The function of histone methylation modification was further explored in signaling cascade and biological functions of Hif‐1, including Hif‐1 nuclear transport, autophagy of HSC, and activation of HSC. It was determined that histone methylation modification of Hif‐1 plays an important role in activation and autophagy of HSC, which provided a new perspective in the development of liver fibrosis.

## Materials and methods

### Cell culture and treatment

LX‐2, human HSC line, was grown in Dulbecco's modified Eagle's medium (DMEM) (SH30022.01; Hyclone, Logan, UT, USA) supplemented with 10% inactivated fetal bovine serum (#0500; Sciencell, Carlsbad, CA, USA) as appropriate. The antibiotics penicillin G (100 U·mL^−1^) and streptomycin sulfate (100 μg·mL^−1^) were added. 100 μmol·L^−1^ CoCl_2_ (C8661; Sigma‐Aldrich, St. Louis, MO, USA) was used to induce cells into hypoxia. Methylthioadenosine (MTA) (1 mm, D5011; Sigma‐Aldrich) was utilized to inhibit histone methylation modification 14, 15. Cells were incubated at 5% CO_2_, 37 °C in a humidified atmosphere (CCL‐170B‐8; ESCO, Singapore).

### Animals

BALB/c female mice, 8 weeks old, were obtained from the Wuhan Institute of Biological Products, Wuhan, China. The experiment was approved by the Committee on Animal Research of Tongji Medical College, Huazhong University of Science and Technology. Mice were randomly divided into two groups: the infected group and the control group. Oncomelania snails infected with *Schistosoma japonicum* were purchased from Hunan Province Institute of Parasitosis Control and Prevention, Yueyang, China. *S. japonicum* cercariae were shed from the snails. Each anaesthetized mouse in the infected group was percutaneously infected with 25 cercariae through the shaved abdomen 5, 16, 17. The mice were sacrificed at 8 weeks postinfection, and samples of liver were collected.

### Genomewide expression chips

Human HSC line, LX2, was cultured in normoxia or treated with 100 μm CoCl_2_ for 8 h. Total RNA was extracted with TRIzol Reagent (15596‐026; Invitrogen, Carlsbad, CA, USA) and further purified using Qiagen RNeasy Mini Kit (217004; QIAGEN, Stockach, Germany) according to manufacturer's instructions. RNA quality was assessed by formaldehyde agarose gel electrophoresis, and RNA was quantitated spectrophotometrically. Genomewide expression chip analysis was performed via technical support from GCBI (Shanghai, China). The samples were processed using Affymetrix GeneChip WT PLUS Reagent Kit (Affymetrix, Carlsbad, CA, USA), followed by Hybridization Wash and Stain Kit. Microarray expression profiles were collected using Affymetrix Human Transcriptome Array 2.0. Original CEL and files were analyzed by Affymetrix software programs Expression Console and Transcriptome Analysis Console. Genes with lower expression in CoCl_2_‐treated cells than in normoxia cells with a fold change > 1.2 (*P* < 0.05) were selected as down‐regulated ones, and those with higher expression in CoCl_2_‐treated cells than in normoxia cells with a fold change > 1.2 (*P* < 0.05) were selected as up‐regulated ones.

### Real‐time PCR

Transcripts of *ogt* were measured by real‐time PCR. Total RNA was isolated from LX‐2 cells by TRIzol Reagent, and 2 μg of RNA was reversely transcribed to cDNA with ReverTra Ace qPCR RT kit (K1622; Thermo, Carlsbad, CA, USA). Gene expression was quantified using FastStart Universal SYBR Green Master (Rox) (04913914001; Roche, Mannheim, Germany) on the real‐time PCR detection system (StepOnePlus™; ABI, Carlsbad, CA, USA). All assays were performed in duplicates for three independent experiments. Specifical primers (Beijing Genomics Institute, China) used in this study were listed as followed: human *ogt*: (forward, 5′‐GTTCCGGCCCATGT TGTTTC‐3′; reverse, 5′‐ACGTTTCGTTGGTTCTGTGC‐3′) and human GAPDH: (forward, 5′‐ACCCAGAAGACTGTGGATGG‐3′; reverse, 5′‐CACATTGGGGTAGGAAC AC‐3′). Relative expression of target gene was analyzed using established ΔΔCt threshold method (ΔCT = CT_Target_ − CT_GAPDH_, ΔΔCT = ΔCT_Test_ − ΔCT_Control_).

### Western blot

Cells were collected at indicated time. Nuclear sample and cell lysates were separated using Nuclear and Cytoplasmic Protein Extraction Kit(P0028; Beyotime, Shanghai, China) and resolved in RIPA lysis buffer (P0013B; Beyotime). Protein concentration was valued using BCA Protein Assay Kit (P0011; Beyotime). Protein samples were then separated by SDS/PAGE and transferred onto polyvinylidene difluoride membrane (PVDF; Millipore, Burlington, MA, USA). After blocking in 5% BSA, membranes were incubated with primary antibodies (Hif‐1α, 1 : 1000, ab16066; Abcam, Cambridge, MA, USA; OGT, 1 : 1000, ab177941; Abcam; H2AFX, 1 : 1000, 10856‐1‐AP; Proteintech, Wuhan, China; H3K4me3, 1 : 1000, 39159, Active Motif, Carlsbad, CA, USA; LC‐3B, 1 : 1000, 12741, Cell Signaling, Danvers, MA, USA; Lamin B1, 1 : 1000, 12987‐1‐AP, Proteintech; α‐SMA, 1 : 1000, ab32575, Abcam; Vimentin, 1 : 1000, EPR3776, Epitomics, Burlingame, CA, USA; β‐actin, 1 : 3000, #M20010; Abmarts, Shanghai, China) and then corresponding secondary antibodies. Immunoreactive bands were tested with SuperSignal™ ELISA Femto Maximum Sensitivity Substrate (37075; Thermo).

### Immunoprecipitation

Cells were lysed in 4 °C precooled RIPA buffer as described above, and 1 mg of cell lysate was incubated with 4 μg Hif‐1α monoclonal antibody (ab16066; Abcam) or H3K4me3 antibody (39159; Active Motif) at 4 °C overnight with continuous agitation. Protein A+G agarose (P2012; Beyotime) was added and incubated for additional 2 h at 4 °C. The beads were washed five times with PBS. Precipitated proteins were eluted by boiling the beads in 2×SDS/PAGE sample buffer for 5 min. The samples were analyzed by western blot with anti‐H3K4me3 or anti‐Hif‐1α antibody.

### Immunohistochemistry

The formalin‐fixed and paraffin‐embedded liver tissues were cut into 4‐μm sections and then deparaffinized routinely. The slides were heated in 10 mm citrate buffer (pH 6.0) for antigen retrieval. After washing with PBS for three times, the slides were incubated with 3% H_2_O_2_ at room temperature for 10 min and then incubated with monoclonal antibody to OGT (ab177941; Abcam) at 4 °C overnight. The slides were washed with PBS and incubated with polyperoxidase‐anti‐rabbit IgG (Envision™, DAKO, Beijing, China) at room temperature for 30 min. After washing, the slides were colored with 3, 3‐diaminobenzidine and counterstained with hematoxylin.

### Immunocytochemistry

LX2 cells were seeded at a density of 3 × 10^5^ cells per well on coverslips. Cells were fixed in 4% paraformaldehyde for 15 min, permeabilized with 0.1% Triton X‐100 in PBS for 10 min, and counterstained with antibodies against vimentin (1 : 100, 2707‐1, Epitomics), Hif‐1α (1 : 100, ab16066; Abcam), P62 (1 : 100, PM045, MBL, USA), or Phalloidin‐Atto 550 (1 : 50, 19083, Sigma‐Aldrich). The coverslips were mounted onto slides in antifade mounting medium (P0126; Beyotime), and fluorescent images were captured (DMI3000B; Leica, Wetzlar, Germany).

### Statistical analysis

All data are expressed as mean ± SD. Differences between experimental and control groups were assessed by one‐way ANOVA using GraphPad Prism 5, *P < 0.05, **P < 0.01.

## Results

### Genes related to histone methylation modification were up‐regulated in hypoxia‐induced LX‐2 cells

**Figure 1:**
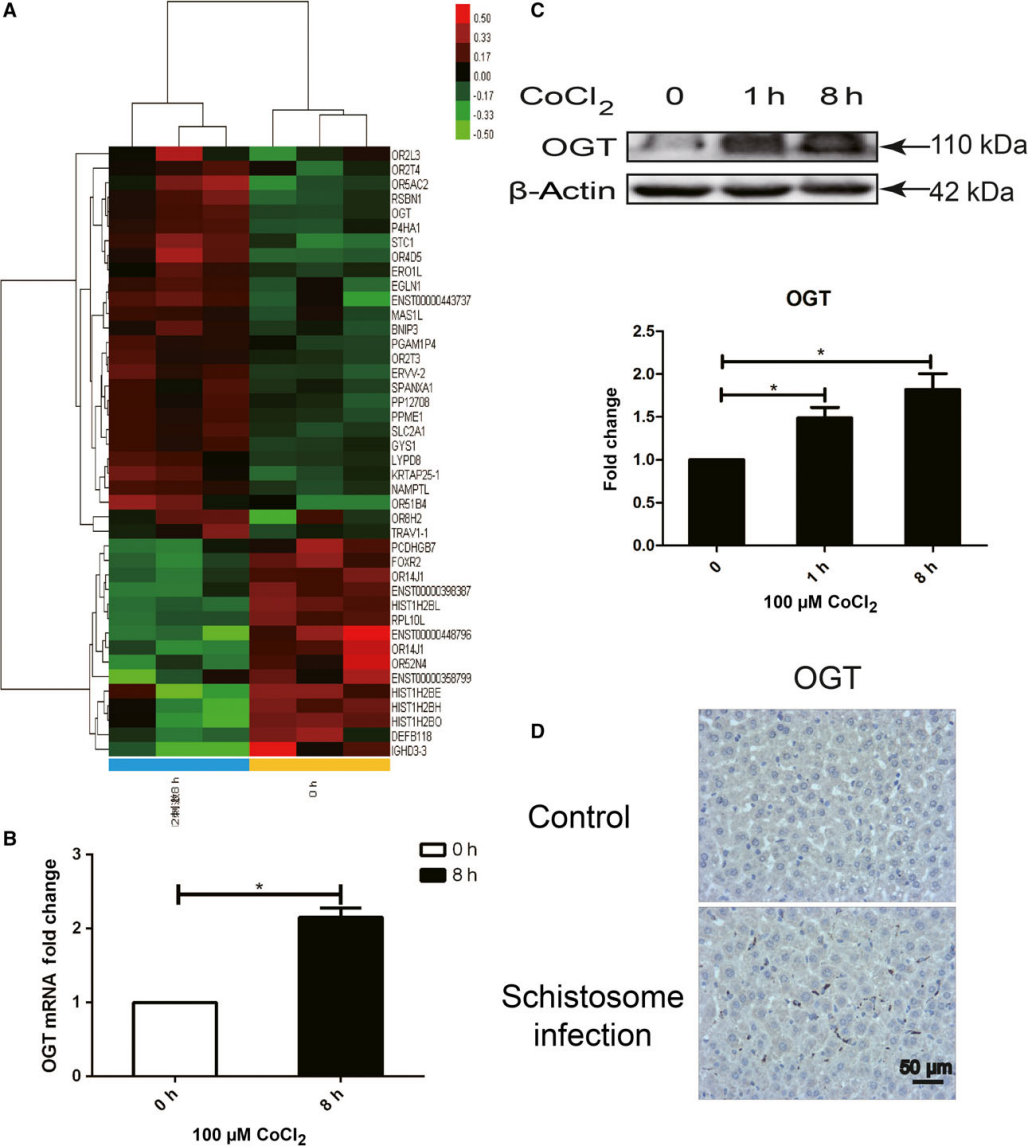
Genes related to histone methylation modification were up‐regulated in hypoxia‐induced LX‐2 cells. Total RNA was extracted and reversely transcribed into cDNA from normal oxygen or CoCl_2_‐treated hypoxia‐induced human hepatic stellate cell line LX‐2. (A) Cluster analysis of genomewide expression chips (red: up‐regulated genes, green: down‐regulated genes). (B) The expression of *Ogt,* a representative up‐regulated gene from genomewide expression chips, was detected at mRNA level by qPCR. Densitometric analysis was performed using pooled data from three such experiments. Data were mean ± SD (*P < 0.05). (C) Total protein was extracted at 0, 1, and 8 h from hypoxia‐induced LX‐2 cells, and the expression of OGT was detected by western blot. Densitometric analysis was performed using pooled data from three such experiments. Data were mean ± SD (*P < 0.05). (D) BALB/c female mice, 6–8 weeks old, were percutaneously infected with 25 cercariae of *Schistosoma japonicum* through the shaved abdomen, sacrificed at 8 weeks postinfection, and samples of liver were collected. The expression of OGT in *S. japonicum*‐infected (*n* = 3) and noninfected (*n* = 3) mice liver was detected with immunohistochemistry and representative images were shown.

We have previously confirmed that Hif‐1, an important transcription factor, affects the activation of HSCs by regulating autophagy. The detailed mechanism needs still further research. Total RNA from normal and CoCl_2_‐pretreated hypoxia‐induced LX‐2 cells was extracted for genomewide expression analysis. Significantly, up‐regulated or down‐regulated genes in hypoxia‐induced LX‐2 cells, compared with cells in control group, were selected and further analyzed (Fig. 1A, Table S1). Among them, GSY1 (glycogen synthase 1), GLUT‐1 (glucose transport protein‐1, also known as SLC2A1), BNIP3 (BCL2/adenovirus E1B 19 kDa interacting protein 3), P4HA1 (prolyl 4‐hydroxylase, alpha polypeptide I), and STC1 (stanniocalcin‐1) were previously determined as Hif‐1 regulated genes 18, 19, 20. However, genes involved in histone methylation modification, such as OGT (O‐linked *N*‐acetylglucosamine (GlcNac) transferase), KDM3A (lysine (K)‐specific demethylase 3A), and KDM2A (lysine (K)‐specific demethylase 2A), were paid attention. OGT, KDM3A, and KDM2A were reported to, respectively, regulate H3K4me3 histone methylation, H3K9me2 histone methylation, and H3K36 de‐methylation 21, 22, 23. The increased expression of *ogt* in hypoxia‐induced LX‐2 was further validated by qPCR at transcriptional level and by western blot at translational level (Fig. 1B,C). In liver section of *S. japonicum‐*infected mice, a recognized animal model of infectious liver fibrosis, increased expression of OGT was also determined with immunohistochemistry (Fig. 1D). Collectively, these results indicated that histone methylation modification might be involved in Hif‐1 signaling cascade during HSC activation.

### H3K4me3 histone methylation modification occurred in Hif‐1 transcriptional complex in hypoxia‐induced LX‐2 human stellate cells

**Figure 2:**
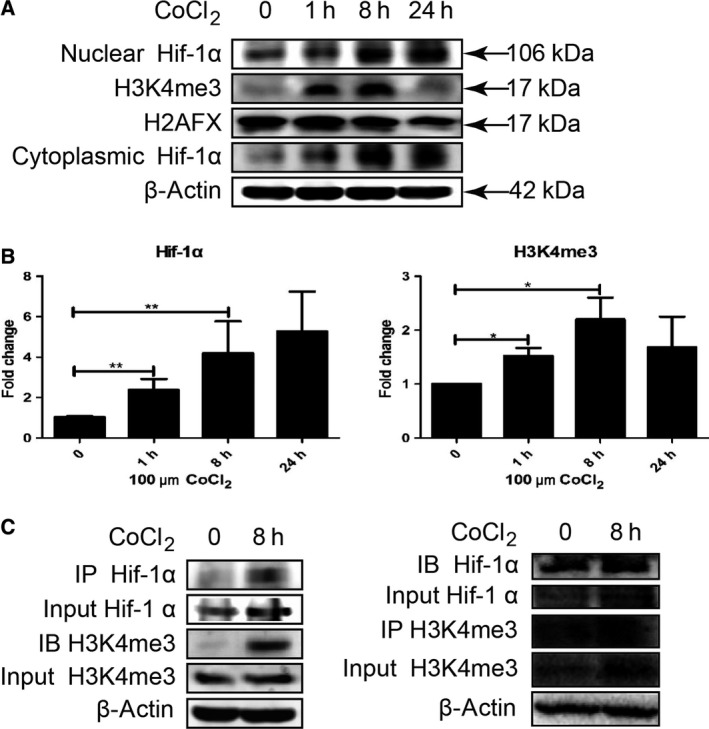
H3K4me3 histone methylation modification occurred in Hif‐1 transcriptional complex in hypoxia‐induced LX‐2 cells. (A) LX‐2 cells were treated with 100 μm CoCl_2._ The cytoplasmic and nuclear protein was extracted at indicated time. Hif‐1α and H3K4me3 were detected by western blot. (B) Densitometric analysis of Hif‐1α and H3K4me3 expression was performed using pooled data from three such experiments. Results were expressed as mean ± SD, *P < 0.05, **P < 0.01. (C) LX‐2 cells were treated with 100 μm CoCl_2_, and 1 mg of cell lysates was collected at indicated time. Anti‐Hif‐1α or anti‐H3K4me3 monoclonal antibody was, respectively, added to cell lysates, and immune‐precipitated proteins were then detected with anti‐H3K4me3 or anti‐Hif‐1α antibody by western blot.

Among histone methylation modification, H3K4me3, trimethylation of histone H3 at lysine 4, was found to exhibit high abundances and especially enrich near the transcriptional start site on promoter region of activated gene. We therefore detected H3K4me3 histone methylation modification in LX‐2 cells under hypoxia using western blot and immunoprecipitation. In nuclear sample, Hif‐1α and histone H3K4me3 was gradually increased as cells were induced with CoCl_2_ (Fig. 2A,B). Consistent with our previous report, in cytoplasm of hypoxic LX‐2 cells, Hif‐1α expression was also increased (Fig. 2A) 5, 6. Anti‐Hif‐1α or anti‐H3K4me3 antibody was, respectively, added to the cell lysate to determine the occurrence of histone H3K4me3 methylation modification in Hif‐1 transcriptional complex with co‐immunoprecipitation. It was shown that H3K4me3 histone methylation modification occurred in Hif‐1 transcription complex in hypoxic LX‐2 cells (Fig. 2C). As a transcriptional regulator, Hif‐1 forms transcriptional complex in cells to exert its biological regulatory function. The above result suggested the formation of Hif‐1 transcription complex in hypoxic LX‐2 cells undergoes histone methylation modification, at least H3K4me3.

### Inhibition of histone methylation modification affects Hif‐1α nuclear translocation in hypoxia‐induced LX‐2 cells

**Figure 3:**
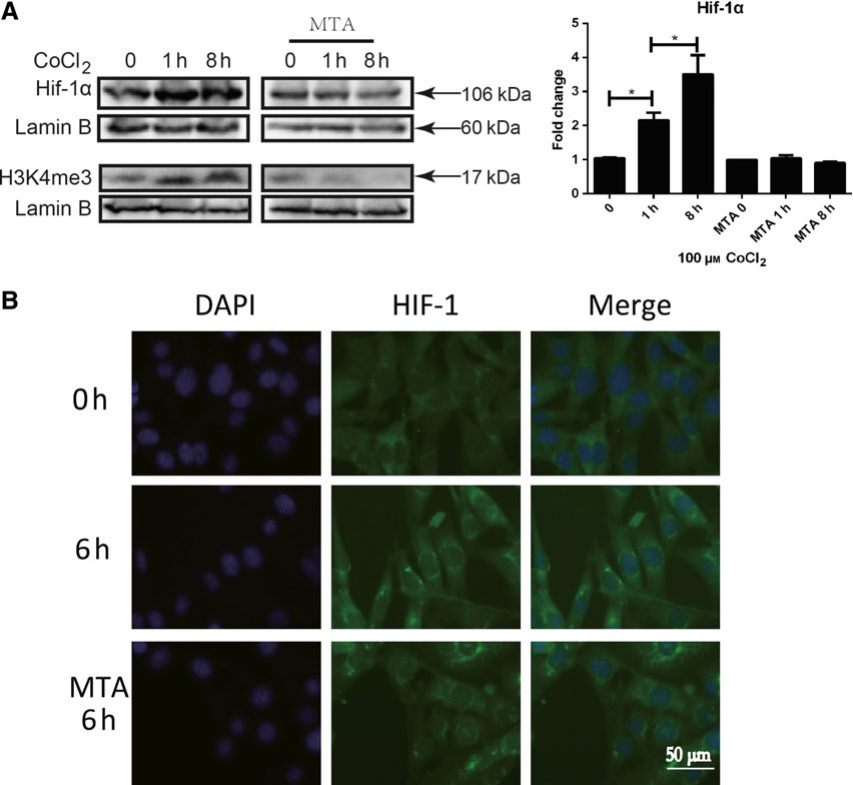
Inhibition of histone methylation modification affects Hif‐1α nuclear translocation in hypoxia‐induced LX‐2 cells. LX2 cells were pretreated with 1 mm MTA (methylation inhibitor) for 16 h and stimulated with 100 μm CoCl_2_. Cells were collected at indicated time. (A) Hif‐1α and H3K4me3 were detected in nuclear extracts by western blot. Densitometric analysis of Hif‐1α expression was performed using pooled data from three such experiments. Results were expressed as mean ± SD, *P < 0.05. (B) Hif‐1α nuclear translocation was detected with immunofluorescence staining. Green: Hif‐1α, blue: DAPI, 400×.

Under hypoxia stimulation, degradation of Hif‐1α was suppressed. Hif‐1α forms heterodimer with Hif‐1β and translocates into the nucleus. Hif‐1 transcription complex binds with promoters of target genes to induce gene expression. To further explore the role of histone methylation modification in Hif‐1 nuclear transport, methylation inhibitor MTA was used to inhibit histone methylation modification 14, 15 in LX‐2 cells and the nucleation of Hif‐1α was detected with western blot and immunofluorescence staining. It was determined that CoCl_2_‐induced hypoxia led to nuclear transport of Hif‐1α, while inhibition of histone methylation modification led to suppress of nuclear translocation of Hif‐1α (Fig. 3A,B). Collectively, the results indicated that in hypoxia‐stimulated HSC, histone methylation plays an important role in Hif‐1α nuclear transport.

### Inhibition of histone methylation modification blocks autophagy in hypoxia‐induced LX‐2 cells

**Figure 4:**
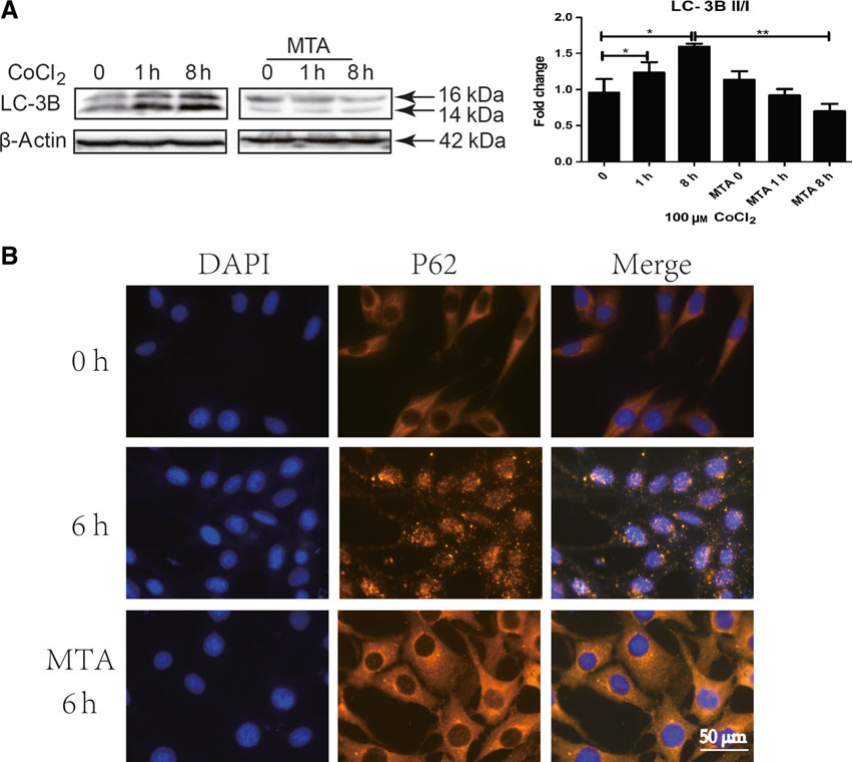
Inhibition of H3K4me3 histone methylation modification blocks autophagy in hypoxia‐induced LX‐2 cells. LX2 cells were pretreated with 1 mm MTA for 16 h and stimulated with 100 μm CoCl_2_. Cells were collected at indicated time. (A) Autophagy marker, LC‐3B, was detected by western blot, and densitometric analysis was performed using pooled data from three such experiments. Results were expressed as mean ± SD, *P < 0.05, **P < 0.01. (B) Autophagy marker, P62 punctate aggregation, was detected with immunofluorescence staining. Yellow: P62, blue: DAPI, 400×.

It was previously reported that Hif‐1 regulates HSCs activation by autophagy. Histone methylation modification was inhibited by MTA, and autophagy markers, LC‐3B and P62, were detected with western blot and immunofluorescence staining. It was shown that hypoxia stimulation led to increase in 14‐kDa lipidated LC‐3B and formation of autophagosome indicated by P62 (Fig. 4A–C), which indicated the occurrence of autophagy. However, as cells were treated with MTA, autophagy induced by hypoxia treatment was significantly inhibited (Fig. 4A–C), which suggested that histone methylation modification contributes to autophagy in LX‐2.

### Deficiency of histone methylation modification impedes the activation of hypoxia‐induced LX‐2 cells

**Figure 5:**
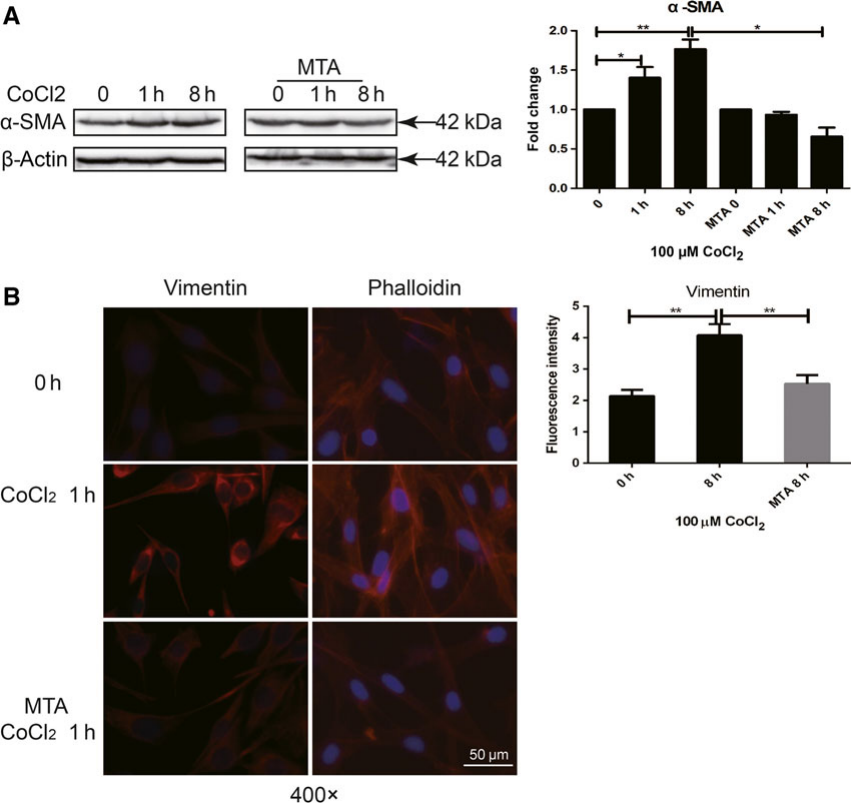
Deficiency of H3K4me3 histone methylation modification impedes the activation of hypoxia‐induced LX‐2 cells. LX2 cells were pretreated with 1 mm MTA for 16 h and stimulated with 100 μm CoCl_2_. Cells were collected at indicated time. (A) Activation of molecular marker of HSC, α‐SMA, was detected by western blot, and densitometric analysis was performed using pooled data from three such experiments. Results were expressed as mean ± SD, *P < 0.05, **P < 0.01. (B) Increase in vimentin (red) and re‐organization of F‐actin (red, CY3‐labeled phalloidin) were detected with immunofluorescence staining (blue: DAPI, 400×).

In order to investigate the relationship between histone methylation modification and activation in hypoxia‐induced LX‐2, we used methylation inhibitor MTA to inhibit histone methylation and observed whether activation of HSC was impacted. LX2 cells were pretreated with 1 mm MTA for 16 h and then stimulated by CoCl_2_. In CoCl_2_‐treated LX2 cells, increase in α‐SMA, vimentin, and cytoskeleton rearrangement indicated the activation of cells (Fig. 5A–C). As CoCl_2_‐treated LX2 cells were pretreated with MTA, the expression of α‐SMA in HSCs was significantly inhibited (Fig. 5A,B). In addition, MTA treatment inhibited vimentin expression and cytoskeleton rearrangement (Fig. 5C), indicating that histone methylation was involved in hypoxia‐induced LX‐2 activation.

## Discussion

Our previous work has reported that hypoxia‐inducible factor 1, Hif‐1, regulates phenotypic transformation and activation of HSCs by autophagy, when the liver is stimulated by inflammation or various factors that form a local hypoxic micro‐environment 5, 6. In recent years, more and more genes were determined as target genes of Hif‐1, including *vegf* (vascular endothelial growth factor), *pgk‐1* (phosphoglycerate kinase 1), *ldha* (lactate dehydrogenase A), and *glut‐1* (glucose transport‐1) 18, 19, 20. Activities of Hif‐1 target genes vary according to different physiological or pathological circumstances. In current work, preliminary study of Hif‐1 potential target genes in HSC was screened using genomewide expression chips. Among differential genes in normoxia and CoCl_2_‐treated hypoxia‐induced LX‐2 cells, *bnip3, p4 ha1, glut1*,* gsy1,* and *stc1* were previously determined as target genes of Hif‐1. Genes such as *ogt*,* kdm3a*, and *kdm2a* were reported to be involved in different forms of histone methylation modification 21, 22, 23. The increased expression of OGT was further confirmed at mRNA and protein level in hypoxia‐induced LX‐2 cells, and also in tissue samples of liver fibrosis infected by *S. japonicum*.

Recently, it was reported that OGT regulates H3K4me3 histone methylation modification 21, 24. OGT (O‐linked *N*‐acetylglucosamine (GlcNAc) transferase) catalyzes the GlcNAc glycosylation of serine/threonine hydroxyl group on the protein surface 25. O‐GlcNAc glycosylation is a special post‐translational modification of proteins 26. OGT regulates the subcellular localization and enzymatic activity of TET3, which converts 5mC to 5‐hydroxymethylcytosine 27, 28. OGT catalyzes the *O‐*GlcNAcylation of TET3 and promotes TET3 nuclear export, which consequently inhibits the formation of 5‐hydroxymethylcytosine catalyzed by TET3 29. Studies have shown that the interaction of TET2 and TET3 promotes the occurrence of H3K4me3 in the promoter region of target genes and enhances expression of corresponding genes. When expression of either TET2/3 or OGT is inhibited, H3K4me3 histone methylation will be suppressed, resulting in a reduction in expression of target genes 21. Furthermore, complex interaction of OGT and Hif‐1 was reported in research of cancer, which indicates that OGT regulates Hif‐1 signaling to catalyze O‐GlcNAcylation reprogramming cancer cell metabolic and survival response 30. In current work, it was firstly determined that OGT increasingly expressed in hypoxia‐induced HSCs and in tissues of liver fibrosis. The detailed role of OGT in Hif‐1 signaling cascade and in development of liver fibrosis is worthy further research.

In this work, research from histone methylation modification was investigated to reveal the mechanism and function of Hif‐1 to HSC activation, as Hif‐1 acts as a master transcriptional factor. H3K4me3, trimethylation of histone H3 at lysine 4, is an important marker of histone methylation modification in chromatin, which is involved in activation of gene expression. As previously reported, hypoxia induces H3K4me3 histone methylation modification in cells 12. We determined that H3K4me3 histone methylation modification occurred in Hif‐1 transcriptional complex in hypoxia‐induced LX‐2 cells. As previously reported, Hif‐1 regulates activation of HSC via autophagy 6. Nuclear transport of Hif‐1α molecule, and autophagy and activation of HSC were apparently inhibited in hypoxia‐induced LX‐2 cells, as H3K4me3 histone methylation was inhibited by MTA, suggesting that histone methylation modification plays an important role in Hif‐1 signaling cascade to regulate cell activities.

Autophagy is an evolutionarily conserved process through autophagosome‐dependent lysosomal degradation of cytoplasmic components, which is essential to scavenge the toxic accumulation of abnormal protein aggregates and organelles, to sustain metabolism, as cells are lack of nutrients and oxygen. As liver is injured, a large number of lipid droplets in HSCs gradually reduced or even disappeared. It is generally believed that autophagy has a crucial function in lipid droplet degradation (known as lipophagy) 31, 32, 33. Studies have shown that epigenetic modifications regulate the occurrence of autophagy at transcriptional and post‐transcriptional level and play an important role in biological functions of autophagy 34. Study of epigenetic modification in autophagy is a hot topic in the current research. The mechanisms of epigenetic modification in Hif‐1 regulating HSC activation via autophagy to degrade lipid droplet remain further research.

In summary, our studies demonstrated that histone methylation modification plays a pivotal role in HSCs autophagy and activation. Inhibition of H3K4me3 histone methylation modification affected Hif‐1 nuclear transport, ultimately affected autophagy and activation of HSCs. Mechanisms of histone methylation modification in Hif‐1 signaling activities, including regulation of autophagy and activation of HSCs, are worthy researched, to deeply understand the mechanism of activation of HSC and development of liver fibrosis, and to provide new therapeutic interventions for liver disease.

## Author contributions

FH and CS conceived and designed the project. FH, LW, and JL performed most of the experiments. KH, ZX, and YZ performed some of the experiments. FH, LW, JL, and CS analyzed and interpreted the data. FH and CS wrote the manuscript. All the authors have read this manuscript.

## Supporting information


**Table S1.** Differential expression gene analysis in hypoxia‐induced and normoxia‐cultured LX‐2 cells.
